# Supplementary material for: Effects of Bafa Wubu and He-Style Tai Chi exercise training on physical fitness of overweight male university students: A randomized controlled trial
Source: PLoS One. 2024 Jan 19;19(1):e0297117. doi: 10.1371/journal.pone.0297117 (PMC10798526; doi:10.1371/journal.pone.0297117)
Supplement: S1 File — (PDF) [file pone.0297117.s001.pdf]

KHON KAEN UNIVERSITY

This is to certify that

The Project Entitled: The Effects of Two Different Types of Tai Chi Exercise Training on Overweight People: A Study on Chinese University Students

Principle Investigator: Mr. Yantao Niu
Faculty of Graduate School, Khon Kaen University

Co- Investigator: Dr. Rojapon Buranarugsa
Faculty of Education, Khon Kaen University

Documents Acceptance:

1. KKUEC Application form, version 3.0, dated 31 July 2021
2. Clinical Trial Protocol, version 3.0, dated 31 July 2021
3. Information Sheet, (Pilot study) English version 3.0, dated 31 July 2021
4. Information Sheet, (Pilot study) Chinese version 3.0, dated 31 July 2021
5. Information Sheet, (Experimental study) English version 3.0, dated 31 July 2021
6. Information Sheet, (Experimental study) Chinese version 3.0, dated 31 July 2021
7. Informed Consent Form, (Pilot study) Chinese version 3.0, dated 31 July 2021
8. Informed Consent Form, (Experimental study) Chinese version 3.0, dated 31 July 2021
9. Study Recruitment Flyer, English version 3.0, dated 31 July 2021
10. Study Recruitment Flyer, Chinese version 3.0, dated 31 July 2021
11. Research Instrument, version 3.0, dated 31 July 2021
12. Investigator's Curriculum Vitae

Record No. 4.2.01: 22/2021
Reference No. HE642132

have been reviewed by the Khon Kaen University Ethics Committee for Human Research based on the Declaration of Helsinki and the ICH Good Clinical Practice Guidelines. **Please submit the renewal report within** June 13, 2022

Date of Approval: 04 August 2021

Date of Expiration: 13 June 2022

(Associate Professor Somdej Pinitsoontorn M.D.)

Chairman of Panel 2

The Khon Kaen University Ethics Committee for Human Research

Office of The Khon Kaen University Ethics Committee in human research

Office of President building 2 Floor 2 nd

Khon Kaen University, 40002 Thailand

Tel.: +66-43-203331, 42942 Fax: +66-43-203331

Office of The Khon Kaen University Ethics Committee in human research (Sub office)

Room 5317, 3rd Floor Wadwichakarn Building, Faculty of Medicine, Khon Kaen University

Mobile 089-7141913 Tel. 67133 – 4

Institutional Review Board Number: IRB00008614

Federal wide Assurance; FWA00003418

Information sheet (Experimental study)

Research topics: The Effects of Two Different Types of Tai Chi Exercise Training on Overweight People: A Study on Chinese University Students

Principle investigator/researcher: YANTAO NIU

Co-researcher: Dr. Rojapon Buranarugsa

Source of funding: No supported research fund

Introduction: Overweight or obesity can affect various functions of the human body, constituting a major public health threat. Tai Chi is a systematic whole-body exercise developed in ancient China that can improve risk factors related to overweight or obesity. The newly compiled simplified Bafa Wubu Tai Chi and He-style Tai Chi have yet to find research on the effects of obese or overweight people.

Objectives: The purpose of this study is to evaluate the effects of two different Tai Chi exercises on the physical fitness and blood lipids of overweight male college students, and the second is to compare the effects of two different Tai Chi exercises on physical fitness and blood lipids.

Participation of research in voluntary: You are cordially invited to participate in our study (2021/9-2021/11). You participated in this research project voluntarily. You are free to decide whether or not to participate in this research project, and after agreeing to participate in the research project. You can withdraw from the research project. Not participating in a research program and withdrawing from it will not affect your current or future academic performance or eligibility for medical care.

Other options if not participating in the program: No need to attend the test.

Procedures: You will be one of 90 volunteer overweight students from three different universities. You will be randomly assigned to 3 groups (simplified Tai Chi group, traditional Tai Chi group and control group), simplified Tai Chi group and traditional Tai Chi group in addition to the normal study and life. Under the guidance of a qualified Tai Chi instructor, the content of the teaching material will be used to conduct Tai Chi intervention exercises for 12 weeks in your free time. The training time will be arranged in the afternoons of Monday, Wednesday and Friday (5:30-6:30), which is just the time for students' daily activities. All assessments are scheduled on Saturdays and Sundays (8:30-11:30 am, 2:30-5: 30 pm). The control group is required to maintain normal activities in addition to normal study and life. Diet, and three health education courses once a month. These will be arranged in spare time. There will be some physical assessments before and after Intervention 12 weeks. The assessment time will be concentrated on Sundays before and after the intervention, which will not affect your normal class and study. If you agree to participate in this project, qualified participants will fill out the Par-Q athletic ability assessment form. All participants will undergo a series of tests

CERTIFIED TRUE COPY ORIGINAL DOCUMENT KKUEC
DATE 04 AUG 2021

before and after the intervention: blood lipid measurement (TC, TG, LDL-C, HDL-C) and physical fitness assessment (weight, BMI, fat percentage, muscle content, flexibility, upper and lower limb strength, balance Sex and aerobic endurance).

Step	Order	Research activities	Time spent	Place
1	1	Receive the research information and sign the consent form	1-2 days	Fitness center
	2	Baseline evaluation (gender, age, height, weight, and BMI). Answer questionnaire (Par-Q athletic ability assessment form)	1-2 days	Fitness center
2	1	Pre-test:<br>1) Blood lipid by nurse using fingertips technique (The blood of the participant will be obtained, the amount is 0.1-0.3ml each time, once before and after 12 weeks.).<br>2)Physical fitness test (Body composition (Body composition analyser), Flexibility (Sit and Reach Test), Upper limb strength (Grip strength test), Lower limb strength (Wall squat test), Balance (Static balance measuring: The Balance Error Scoring System (BESS), Dynamic balance measuring: The Star Excursion Balance Test (SEBT)), Aerobic endurance (6MWT),)	1-2 days	1)Jaozuo People's Hospital<br>2)Tai Chi Sports Fitness Testing Center
3	1	Random assignment (The eligible participants will be randomly allocated in a 1:1:1ratio to either the Bafa Wubu Tai Chi group, tradition He-styles Tai Chi group and control group. The random allocation sequence will be generated using web programming at http://www.randomizer.org)	1-2 days	Fitness center
	2	Tai Chi program for participants<br>1) 15 actions of the static stretching for warm up and cold down. It should be the same as Experiment study group. total time is 30 min.<br>2) The 90 participants will be randomly divided the subjects into three groups: Bafa Wubu Tai Chi Group (BW-TCG) and Traditional He-style Tai Chi Group (TH-TCG) and control group. The BW-TCG group and the TH-TCG group will have 3 courses three times a week (5:30-6:30 p.m., Monday, Wednesday and Friday) for 12 weeks under the guidance of a Tai Chi qualified instructor during extracurricular activity time, in addition to their normal study and life. The control group is required to maintain normal activities in addition to normal study and life. Diet, and three health education courses once a month. These will be arranged in spare time.	12 weeks	Tai Chi Hall of Jiaozuo Tai Chi Sports Center
4	1	Post-test<br>In this part, the test content as same as Pre-test.	1-2 days	Same as the pre-test place

Risk and discomfort: If you participate in this study, you may suffer some sports injuries, for example muscle soreness, joints (knee or ankle) pain due to some-incorrect posture of Tai Chi during exercise. However, primary-health care (such as ice pack, pain killer, etc.) will be prepared by the nurse of our research group.

Benefits/Direct benefits: According to results from previous studies in overweight and obesity, if you participate in this study, you will gain Tai Chi skills, increase courage, perseverance and feel the joy of Tai Chi. At the same time, it may improve your physical fitness or improve your health.

The cost of research/offset travel/time cost: Each volunteer in the control group: 20 Yuan/time × 2 times = 40 Yuan. You will receive 20 Yuan each time you participate. Each volunteer in the intervention group: 5 Yuan/time×36 times=180 yuan. You will receive 5 Yuan each time you participate. If you have to travel to participate in the research site, you have to receive the travel expenses the same amount as you have paid. You will receive it every time you participate in research project.

Confidentiality: The researchers will keep the data of each volunteer confidential, and we will use the ID of each volunteer to record the data and access the data. We will write research

CERTIFIED TRUE COPY OF ORIGINAL DOCUMENT
KKUEC
DATE 04 AUG 2021

reports or publish papers in journals after the research is over, but we will not use your name in the reports. At the end of the study, the basic personal data of all participants will be destroyed by the shredder. Under no circumstances will the basic information be made public. This research only saves and analyzes the collected data for research use until the end of the project.

Covid-19 regulations:

Although the epidemic prevention and control situation in China is good, our testers have been vaccinated, and the object of our research is the overweight students (vulnerable groups) in the school. Although these college students have been vaccinated, when we provide guidance and intervention, we will Follow the COVID-19 guidelines, show the health QR code, wear a mask, wash your hands with hand sanitizer, and maintain social distancing as much as possible. Although it is unlikely that schools will be temporarily closed again, we have prepared contingency plans, such as postponing trials.

Whom to contact:

Please feel free to participate in this course. If you have questions, please contact any of the following persons:

1) Mr. YANTAO NIU

Address: Faculty of Sports College of Jiaozuo Normal college at 998 Shanyang Road, Jiaozuo City, 454000, Tel:13782718127

2) Assistant Professor Dr. Rojapon Buranarugsa

Address: Faculty of Education 123 Moo 16 Mittraphap road Mueng Khon Kaen District, Khon Kaen, Thailand, 40002, Tel: +66909963752

If you have been treated incorrectly, or you want to know your rights while participating in this research, please contact:

1) Center for Ethics in Human Research, Khon Kaen University

Address: Office of President building 2 Floor 2nd Khon Kaen University, Mittraphap road, Nai Muang Subdistrict, Muang District, Khon Kaen, 40002 Thailand Tel. +6689-7141177

2) The Scientific Research Office of Jiaozuo Normal college

Address: The Scientific Research Office of Jiaozuo Normal college at 998 Shanyang Road, Jiaozuo City. Tel: 0391-3589401

CERTIFIED TRUE COPY OF AN ORIGINAL DOCUMENT
DATE 04 AUG 2021

信息表单（试验研究）

研究题目：两种不同类型太极拳运动训练对超重人群的影响:一项对中国大学生的研究

研究者：牛炎涛

共同研究者：Dr. Rojapon Buranarugsa

资金来源：没有资金支持（自费）

介绍：超重或肥胖可影响人体的各种功能，构成重大的公共卫生威胁。太极拳是中国古代发展起来的一种系统的全身运动，可以改善与超重或肥胖相关的危险因素。新编制的简化的八发五步太极拳和和式太极拳尚未发现肥胖或超重的人的影响的研究。

研究目的：本研究的目的是评价两种不同太极拳运动对超重男大学生体质和血脂的影响，二是比较两种不同太极拳运动对体质和血脂的影响。

自愿参与研究：诚挚邀请您参与我们的研究（2021/9-2021/11）。您自愿参与了这个研究项目，您可以自由决定是否参与该研究项目，并在同意参与该研究项目后，您可以退出研究项目，不参与研究计划和退出研究计划不会影响您当前或未来的学业成绩或医疗保健资格。

不参加该计划的其他选择： 无需参加测试。

研究程序：您将成为来自三所不同大学的 90 名超重学生志愿者之一。除了正常的学习和生活，你将被随机分配到 3 组（简化太极组、传统太极组和对照组）。简化太极组和传统太极组，在有资质的太极教练指导下，利用教材内容，在空闲时间进行 12 周的太极干预练习。培训时间安排在周一、周三和周五的下午（5:30-6:30），正好是学生日常活动的时间。所有评估都安排在周六和周日（上午 8:30-11:30，下午 2:30-5:30）。对照组除正常学习和生活外，还需保持正常活动和饮食，每月一次共三次健康教育课程。这些将在空闲时间安排。在干预 12 周前后会有一些身体评估。评估时间集中在干预前后的周日，不影响正常上课和学习。如果您同意参加此项目，合格的参与者将填写 Par-Q 运动能力评估表。所有参与者将在干预前后进行一系列测试：血脂测量（TC、TG、LDL-C、HDL-C）和体能评估（体重、BMI、脂肪百分比、肌肉含量、柔韧性、上下限）四肢力量、平衡性和有氧耐力）。

阶段	步骤	研究行为内容	花费时间	地点
1	1	接收研究资料并签署同意书	1-2 天	健身中心
	2	回答问卷（Par-Q 运动能力评估表	1-2 天	健身中心
2	1	干预前测试：<br>1) 护士指尖血脂（采集参与者的血液，每次 0.1-0.3ml，12 周前后各一次）。<br>2）体能测试（身体成分（身体成分分析仪），柔韧性（坐姿和伸展测试），上肢力量（握力测试），下肢力量（墙蹲测试），平衡（静态平衡测量：平衡误差评分）系统（BESS），动平衡测量：星际平衡测试（SEBT）），有氧耐力（6MWT），）	1-2 天	1）焦作市人民医院<br>2)太极运动体能测试中心

CERTIFIED TRUE COPY OF AN ORIGINAL DOCUMENT
DATE 04 AUG 2021

3	1	随机分配（符合条件的参与者将按 1:1:1 的比例随机分配到八法五步太极组、传统和式太极组和对照组。随机分配序列将使用 http: 的网络编程生成： //www.randomizer.org)	1-2 天	健身中心
	2	参加者太极拳课程<br>1) 15 个静态拉伸动作，用于热身和冷身。它应该与实验研究组相同。总时间为 30 分钟。<br>2) 90 名参与者将被随机分为三组：八法五步太极组（BW-TCG）和传统和式太极组（TH-TCG）和对照组。BW-TCG 组和 TH-TCG 组在课外活动期间，在一名太极拳合格教练的指导下，每周 3 次（下午 5:30-6:30，周一、周三和周五）进行 3 次课程，为期 12 周时间，除了他们正常的学习和生活。对照组除正常学习和生活外，还需保持正常活动。饮食和每月一次的三个健康教育课程。这些将在空闲时间安排。	12 周	焦作太极体育中心太极馆
4	1	干预后测量<br>本部分测试内容同干预前。	1-2 天	与以上测量地点相同

风险和不适：如果您参与这项研究，您可能会因运动时太极拳的某些不正确姿势而遭受一些运动损伤，例如肌肉酸痛、关节（膝盖或脚踝）疼痛。但是，初级保健（如冰袋、止痛药等）将由我们研究组的护士准备。

好处/直接益处：根据以往超重和肥胖的研究结果，如果您参加这项研究，您将获得太极技能，增加勇气、毅力，感受太极的乐趣。同时，它可能会改善您的身体素质或改善您的健康状况。

研究费用/抵消差旅费/时间费用：对照组每位志愿者：20 元/次×2 次=40 元。每次参加您将获得 20 元。干预组每位志愿者：5 元/次×36 次=180 元。每次参加您将获得 5 元。如果你必须出差参加研究站点，你会收到的相同金额支付您的差旅费。每次参与研究项目时都会收到它。

保密：研究人员将对每位志愿者的数据保密，我们将使用每位志愿者的 ID 来记录数据和访问数据。研究结束后我们会写研究报告或在期刊上发表论文，但我们不会在报告中使用您的名字。在研究结束时，所有参与者的基本个人数据将被粉碎机销毁。在任何情况下都不会公开基本信息。本研究仅保存和分析收集到的数据以供研究使用，直至项目结束。

Covid-19 规定：虽然中国的疫情防控形势良好，但我们的检测人员已经接种了疫苗，我们研究的对象是学校的超重学生（弱势群体）。尽管这些大学生已经接种了疫苗，但在我们提供指导和干预时，我们将遵循 COVID-19 指南，出示健康二维码，戴口罩，用洗手液洗手，并尽可能保持社交距离。虽然学校不太可能再次暂时关闭，但我们已经准备了应急计划，例如推迟试验。

联系方式：请随时参加本课程。如有疑问，请联系以下人员，

1) 牛炎涛先生

地址：焦作市山阳路 998 号焦作师范学院体育学院 454000 电话：13782718127

2) 助理教授 Rojapon Buranarugsa 博士

CERTIFIED TRUE COPY OF AN ORIGINAL DOCUMENT
KKUEC
DATE 0 4 AUG 2021

地址：教育学院 123 Moo 16 Mittraphap road Mueng Khon Kaen District, Khon Kaen, Thailand, 40002, 电话：+66909963752

如果您在参与本研究时受到了不正确的对待，或者您想了解自己的权利，请联系：

1）孔敬大学人类研究伦理中心

地址：Khon Kaen Muang 区 Nai Muang 街道 Mittraphap 路 2 楼 2 楼孔敬大学校长办公室 40002 泰国电话。 +6689-7141177

2）焦作师范学院科研处

地址：焦作市山阳路 998 号焦作师范学院科研处。电话：0391-3589401

CERTIFIED TRUE COPY OF AN ORIGINAL DOCUMENT

DATE 04 AUG 2021

志愿参加同意书

我（先生，小姐）________，年龄____，学校________，院系________，专业________，获得的解释（通知人）________，这是一个“两种不同类型太极运动训练对超重人群的影响：一项对中国大学生的研究”的项目，我是一名志愿者，我已经了解研究项目的详细信息。

-我必须遵循的程序和程序

-我将获得的好处

-参加该计划可能引起的副作用或危险。（根据项目的性质适当指定）

如果愿意，我可以随时退出这种试验，不失去任何权利。如果有副作用或不适我将立即向当时正在训练中的教练或工作人员报告。（在医疗情况下注明）

我已经阅读并理解了上面的解释。因此，已签署同意成为该研究项目的志愿者

自愿者签名：

年/月/日：

通知人签名：

年/月/日：

首席研究员签名：

CERTIFIED TRUE COPY OF AN ORIGINAL DOCUMENT
DATE 04 AUG 2021

年/月/日：

注意：

（1）如果志愿者是一个较大的孩子但未满 18 岁，您可以自己决定。由志愿者（孩子）和父母双方签名。

（2）主治医生不得是自愿同意书，但是可以给出信息/解释。

（3）如果志愿者无法可以阅读/签名的信，改为使用图章，如下所示：

我看不懂这本书。但是研究人员会先阅读我的同意书，直到我理解为止，所以我愿意在这份同意书上按上指纹。

指纹

解释者签名：

见证人：

（一定不能是医生或研究员）

日 期：　　年　　月　　日

两种不同类型太极拳运动训练对超重人群的影响:对中国大学生的研究志愿参加招聘信

时间：2021.7-2021.9

【招募目的】

本研究旨在评价两种太极(八法五步vs传统和式)的效果

【招募条件】

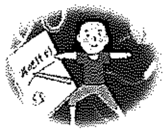

*年龄18-23岁

*大学一、二年级的男大学生

*24≤BMI<27.9(中国超重标准)

【排除标准】

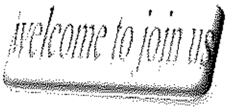

*定期长期的太极拳运动员

*有体育协会会员

*严重的心血管疾病或肌肉骨骼系统疾病

【试验方法】

在一定训练程序指导下进行两种类型太极拳练习。

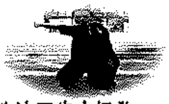

八法五步太极拳

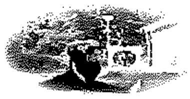

传统和式太极拳

【干预时间】

每周3次，每次1个小时，12周的练习时间，时间会根据同学的上课情况进行调整。

【联系方式】

牛炎涛，体育学院教师，13782718127；

焦作市山阳区998号焦作师范高等专科学校体育学院。

Effects of Excising Two Different Types of Tai Chi on the Overweight: A Volunteer Recruitment Letter of Study on Chinese College Students

Period: 2021.7- 2021.9

[Recruitment Objective]

This study aims to evaluate the effects of two types of Tai Chi (Bafa Wubu vs Traditional He-style

[Requirements]

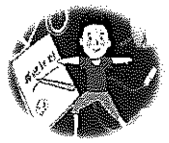

*18-23 years old

* Male freshmen and sophomores

* 24≤BMI<27.9 (China Overweight Standard)

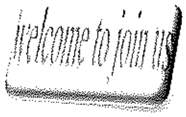

[Exclusion Criteria]

* Regular and long-term Tai Chi players

*Members of sports associations

*With serious cardiovascular or musculoskeletal disease

[Test Method]

Conduct two types of Tai Chi excises under the guidance of a certain training procedure.

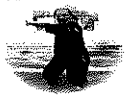

Bafa Wubu Tai Chi

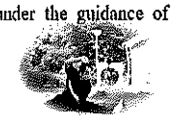

Traditional He-style Tai Chi

[Intervention Time]

3 times a week with 1 hour each time for 12 weeks. Subject to the training situation of the trainees.

[Contacts]

Yantao Niu, Teacher of Physical Education Institute, 13782718127;

Physical Education Institute, Jiaozuo Normal College, 998 Shanyang District, Jiaozuo City.

两种不同类型太极拳运动训练对超重人群的影响:对中国大学生的研究志愿参加招聘信

时间：2021. 7-2021. 9

【招募目的】

本研究旨在评价两种太极（八法五步vs传统和式）的效果。

【招募条件】

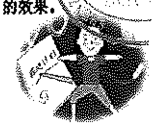

*年龄18-23岁

*大学一、二年级的男大学生

*24≤BMI<27. 9(中国超重标准)

【排除标准】

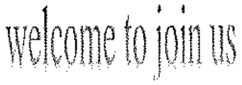

*定期长期的太极拳运动员

*有体育协会会员

*严重的心血管疾病或肌肉骨骼系统疾病

【试验方法】

在一定训练程序指导下进行两种类型太极拳练习。

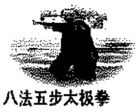

八法五步太极拳

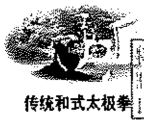

传统和式太极拳

特此声明，此翻译件与原件内容一致
[illegible] IS IDENTICAL TO THE ORIGINAL

【干预时间】

每周3次，每次1个小时，12周的练习时间，时间会根据同学的上课情况进行调整。

【联系方式】

牛炎涛，体育学院教师，13782718127；

焦作市山阳区998号焦作师范高等专科学校体育学院。

Effects of Excising Two Different Types of Tai Chi on the Overweight: A Volunteer Recruitment Letter of Study on Chinese College Students

Period: 2021.7- 2021.9

[Recruitment Objective]

The study aims to evaluate the effects of two types of Tai Chi(Babu Wufa and Traditional He-style).

[Requirements]

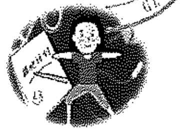

*18-23 years old

* Male freshmen and sophomores

* 24≤BMI<27.9 (China Overweight Standard)

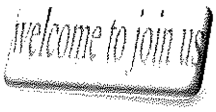

[Exclusion Criteria]

* Regular and long-term Tai Chi players

*Members of sports associations

*With serious cardiovascular or musculoskeletal disease

[Test Method]

Conduct two types of Tai Chi excises under the guidance of a certain training procedure.

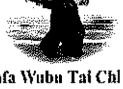

Bafa Wubu Tai Chi

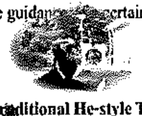

Traditional He-style Tai Chi

[Intervention Time]

3 times a week with 1 hour each time for 12 weeks. Subject to the training situation of the trainees.

[Contacts]

Yantao Niu, Teacher of Physical Education Institute, 13782718127;

Physical Education Institute, Jiaozuo Normal College, 998 Shanyang District, Jiaozuo City.

COPY OF AN ORIGINAL DOCUMENT

DATE 04 AUG 2021

ข้อปฏิบัติสำหรับผู้วิจัย
ในโครงการที่ผ่านการรับรองจริยธรรมการวิจัยในมนุษย์

คณะกรรมการจริยธรรมการวิจัยในมนุษย์มหาวิทยาลัยขอนแก่นแจ้งให้ทราบเกี่ยวกับหน้าที่และความรับผิดชอบของผู้วิจัยภายหลังจากโครงการวิจัยในมนุษย์ได้ผ่านการรับรองด้านจริยธรรมการวิจัยแล้ว ดังต่อไปนี้

1 ผู้วิจัยจะต้องดำเนินการวิจัยตามขั้นตอนต่างๆที่ระบุไว้ในโครงร่างการวิจัยโดยเคร่งครัด โดยใช้เอกสารคำชี้ แจงและแบบยินยอม รวมถึงเอกสารอื่นๆที่ได้ผ่านการรับรองจากคณะกรรมการแล้วเท่านั้น

2 ผู้วิจัยที่มีหน้าที่รายงานต่อคณะกรรมการจริยธรรมฯ ตาม SOP บทที่ 6 เมื่อ

2.1 มีการดำเนินงานวิจัยครบระยะเวลาหนึ่ง <u>ซึ่งจะต้องมีการรายงานความก้าวหน้าตามระยะเวลาที่คณะ กรรมการกำหนดในเอกสารรับรอง</u> หรือเมื่อครบหนึ่งปีจากวันที่ระบุไว้ในเอกสารรับรองจริยธรรม การวิจัยของโครงการ โดยใช้<u>แบบรายงานความก้าวหน้า</u> (KKUEC-Progress, AF/01-06-03.6)

2.2 มีการดำเนินการวิจัยไม่ทันตามที่กำหนด โดยทั่วไปคณะกรรมการจะให้การรับรองไม่เกิน 1 ปี ก่อนวันหมดอายุตามที่กำหนดไว้ในหนังสือรับรอง ผู้วิจัยจะต้องเสนอเอกสารขอต่ออายุการรับรอง โครงการวิจัย โดยใช้ <u>แบบเสนอขอต่อการรับรองโครงการ</u> (KKUEC-Renew, AF/02-06/03.6) ภายใน 30 วันก่อนหมดอายุ เพื่อให้ทางสำนักงานได้มีระยะเวลาจัดเตรียมเอกสารเข้าประชุมก่อนโครงการวิจัยจะหมดอายุ ทั้งนี้หากท่านยังไม่ได้รับเอกสารรับรองการต่ออายุจากคณะกรรมการฯ จะไม่สามารถรับอาสาสมัครใหม่ระหว่างที่โครงการวิจัยหมดอายุได้

2.3 มีความจำเป็นในการปรับปรุงโครงการวิจัย (protocol amendment) หรือ มีการเปลี่ยนแปลงหัวหน้าโครงการวิจัย/เพิ่มเติมผู้ร่วมวิจัย ผู้วิจัยจะต้องเสนอการปรับปรุงเป็น<u>แบบรายงานขอการปรับปรุงโครงการวิจัย</u> (KKUEC-Amend, AF/03-06/03.6) ตามที่ได้กำหนดไว้ โดยอ้างอิงหมายเลขสำคัญโครงการที่ได้รับไว้ โดยต้องระบุให้ชัดเจนว่า มีการเปลี่ยนแปลงอะไร, อย่างไร และเหตุผลที่ต้องมีการเปลี่ยนแปลง ทั้งนี้ในกรณีการเปลี่ยนแปลงหัวหน้าโครงการวิจัย/เพิ่มเติมผู้ร่วมวิจัย คนใหม่ให้แนบประวัติมาด้วย

2.4 มีอาการไม่พึงประสงค์รุนแรงจากการดำเนินโครงการวิจัย (Serious adverse events) เกิดขึ้นแก่อาสาสมัครของโครงการ ผู้วิจัยจะต้องมีเอกสารแจ้งกรรมการภายใน 7 วันปฏิทิน <u>และหากอาการไม่พึงประสงค์รุนแรงนั้นเป็นเหตุให้อาสาสมัครถึงแก่ชีวิต ภายใน 24 ชั่วโมง (โดยทางจดหมาย จดหมายอิเล็กทรอนิกส์ หรือโทรสาร)</u> หลังจากผู้วิจัยทราบเหตุการณ์ โดยใช้<u>แบบรายงานเหตุการณ์ไม่พึงประสงค์สำหรับอาสาสมัครในสถาบัน</u> (KKUEC-SAE-Local, AF/04-06/03.6) และแนบรูปแบบเอกสารรายงานเป็นสำเนา SAE report form ที่กำหนดโดยผู้สนับสนุนทุนวิจัย หากไม่มีแบบรายงานจากผู้สนับสนุนทุกวิจัยให้ใช้แบบรายงานของสำนักงานตามที่กำหนดอย่างเดียว กรณีเป็นรายงานเหตุการณ์ไม่พึงประสงค์ที่เกิดแก่อาสาสมัครนอกสถาบัน ซึ่งบริษัทผู้สนับสนุนส่งให้ผู้วิจัย ให้ใช้<u>แบบรายงานเหตุการณ์ไม่พึงประสงค์ที่เกิดแก่อาสาสมัครนอกสถาบัน</u> (KKUEC-SAE-External, AF/05-06/03.6) แนบกับแบบรายงานเหตุการณ์ไม่พึงประสงค์ที่บริษัทผู้สนับสนุน

2.5 มีการดำเนินการใดๆที่ไม่ถูกต้องตามระเบียบการวิจัยที่กำหนดไว้ ผู้วิจัยจะต้องรายงานให้คณะกรรมการรับทราบภายใน 7 วันปฏิทินหลังจากที่ตรวจพบ โดยใช้แบบ<u>รายงานการดำเนินงานวิจัยที่เบี่ยงเบน</u> (KKUEC-deviation, AF/06-06/03.6)

2.6 การวิจัยเสร็จสิ้นลงหรือยุติการวิจัยด้วยใดๆ ให้ผู้วิจัยมีหนังสือแจ้งปิดโครงการวิจัยนั้นพร้อมผลการดำเนินการวิจัยให้คณะกรรมการทราบตามแบบ<u>รายงานแจ้งการปิดโครงการวิจัย</u> (KKUEC-Close, AF/07-06/03.9)

3 คณะกรรมการจะมีการสุ่มเข้าตรวจเยี่ยมโครงการวิจัยเพื่อตรวจดูความเรียบร้อยของการดำเนินงาน และรับฟัง และให้คำปรึกษาข้อปัญหาที่อาจมีในระหว่างการดำเนินการวิจัย โดยสำนักงานจะมีหนังสือแจ้งให้ทราบ ล่วงหน้าเป็นเวลา 2 สัปดาห์ ผลการตรวจเยี่ยมโครงการวิจัยจะแจ้งเพื่อทราบในที่ประชุมคณะกรรมการ และจะแจ้งผลการพิจารณาให้ผู้วิจัยได้ทราบและอาจมีข้อเสนอแนะนำให้ปฏิบัติต่อไป

ประกาศมหาวิทยาลัยขอนแก่น

(ฉบับที่ ๕๑๗ /๒๕๖๓)

เรื่อง แนวทางการดำเนินการวิจัยในมนุษย์ในช่วงที่มีการระบาด

ของโรคติดเชื้อไวรัสโคโรนาสายพันธุ์ใหม่ ๒๐๑๙ (COVID-19) ฉบับที่ ๒

ตามที่มหาวิทยาลัยขอนแก่นได้ออกประกาศมหาวิทยาลัยขอนแก่น ฉบับที่ ๕๙๒/๒๕๖๓ เรื่อง แนวทางการดำเนินการวิจัยในมนุษย์ในช่วงที่มีการระบาดของโรคติดเชื้อไวรัสโคโรนาสายพันธุ์ใหม่ ๒๐๑๙ (COVID-19) ลงวันที่ ๒๔ มีนาคม ๒๕๖๓ เพื่อให้การดำเนินการวิจัยในมนุษย์ที่ผ่านการรับรองจากคณะกรรมการจริยธรรมการวิจัยในมนุษย์ มหาวิทยาลัยขอนแก่น ดำเนินการโดยคำนึงถึงความปลอดภัยของอาสาสมัคร และสอดคล้องกับสถานการณ์ปัจจุบันนั้น เนื่องจากบัดนี้สถานการณ์การระบาดของโรค COVID-19 สามารถควบคุมได้ดีในระดับหนึ่ง ดังนั้นเพื่อให้การวิจัยในมนุษย์ที่ดำเนินการในมหาวิทยาลัยขอนแก่นสามารถดำเนินการต่อไปได้ อาศัยอำนาจตามมาตรา ๓๗ (๑) แห่งพระราชบัญญัติมหาวิทยาลัยขอนแก่น พ.ศ. ๒๕๕๘ จึงเห็นควรให้ออกประกาศเพื่อให้ผู้วิจัยสามารถดำเนินการวิจัยได้แต่ยังต้อง มีมาตรการในการป้องกันการแพร่กระจายเชื้อดังต่อไปนี้

ข้อ ๑ ผู้วิจัยต้องดำเนินการวิจัยโดยคำนึงถึงความปลอดภัย และสวัสดิภาพของอาสาสมัคร และมีมาตรการป้องกันการระบาดของเชื้อไวรัสโคโรนาสายพันธุ์ใหม่ ๒๐๑๙ โดยยึดหลักการเว้นระยะห่างทางสังคม การสวมหน้ากากอนามัย การรักษาสุขอนามัยส่วนบุคคล การจำกัดการเดินทางที่ไม่จำเป็น และการคัดกรองโรคตามแนวทางมาตรฐาน

ข้อ ๒ สำหรับโครงการวิจัยซึ่งผ่านการรับรองจากคณะกรรมการจริยธรรมการวิจัยในมนุษย์แล้วขอให้ผู้วิจัยดำเนินการดังนี้

๒.๑ สามารถรับอาสาสมัครรายใหม่ได้ โดยผู้วิจัยต้องจัดหามาตรการเพื่อปกป้องอาสาสมัครจากการติดเชื้อไวรัสโคโรนาสายพันธุ์ใหม่ ๒๐๑๙ โดยยึดหลักการเว้นระยะห่างทางสังคม และการคัดกรองโรคตามแนวทางมาตรฐาน

๒.๒ สามารถนัดติดตามอาสาสมัครที่อยู่ในโครงการวิจัยได้ โดยยึดหลักการเว้นระยะห่างทางสังคม และการคัดกรองโรคตามแนวทางมาตรฐาน และให้ลดการติดตามที่อาสาสมัครต้องมีการเดินทางข้ามจังหวัด โดยปรับเปลี่ยนแนวทางการติดตามโดยใช้สื่อออนไลน์ และการให้อาสาสมัครตรวจเลือด ตรวจทางรังสีวิทยา หรือการตรวจอื่น ๆ ที่สถานพยาบาลใกล้บ้านตามความเหมาะสม

๒.๓ สำหรับการวิจัยทางคลินิก อนุญาตให้มีการนัดตรวจติดตามงานวิจัยที่สถาบัน (on-site monitoring) แต่ยังคงมาตรการรักษาระยะห่างทางสังคมและสุขอนามัยส่วนตัวและส่วนรวม โดยผู้ประสานงานโครงการวิจัยของบริษัทจะต้องผ่านการตรวจคัดกรองอุณหภูมิร่างกาย และสวมหน้ากากอนามัยหรือหน้ากากผ้าตลอดเวลาตามระเบียบข้อกำหนดของสถาบัน

๒.๔ สำหรับโครงการวิจัยที่จำเป็นต้องมีการสื่อสารกันระหว่างผู้ประสานงานวิจัยของบริษัทผู้สนับสนุนกับทีมวิจัยของมหาวิทยาลัยขอนแก่น ให้เป็นไปตามมาตรการที่มหาวิทยาลัยกำหนด ทั้งนี้ให้บริษัททำบันทึกถึงคณบดีคณะวิชาที่เกี่ยวข้องเพื่อพิจารณาอนุมัติ

๒.๕ กรณีที่ผู้ตรวจสอบโครงการวิจัย (auditor) ผู้ประสานงานโครงการวิจัย หรือเจ้าหน้าที่อื่นๆ ของบริษัทผู้สนับสนุน เดินทางมาจากต่างประเทศ หรือจากพื้นที่ในประเทศที่มีการระบาด ก่อนเข้ามาดำเนินกิจกรรมในมหาวิทยาลัยขอนแก่น ได้จะต้องผ่านการกักตัวเป็นเวลา ๑๔ วัน หรือดำเนินการตามมาตรการของทางราชการในขณะนั้น

๒.๖ การดำเนินการในโครงการวิจัยที่ปรับเปลี่ยนเพราะสถานการณ์ระบาดของโรคติดเชื้อไวรัสโคโรนาสายพันธุ์ใหม่ ๒๐๑๙ หากไม่มีการแก้ไขในตัวโครงร่างการวิจัย (protocol) ให้ผู้วิจัยรายงานต่อคณะกรรมการจริยธรรมการวิจัยในมนุษย์มหาวิทยาลัยขอนแก่นเป็นเรื่องแจ้งเพื่อทราบโดยไม่ต้องเสนอขอปรับปรุงโครงร่างการวิจัย (protocol amendment)

ข้อ ๓ เนื่องจากสถานการณ์การระบาดของไวรัสโคโรนาสายพันธุ์ใหม่ ๒๐๑๙ นั้นสามารถเปลี่ยนแปลงได้อย่างรวดเร็ว ขอให้ผู้วิจัยดำเนินการวิจัยโดยคำนึงถึงสถานการณ์ โดยหากการระบาดมีแนวโน้มเพิ่มขึ้น ขอให้ผู้วิจัยดำเนินกิจกรรมวิจัยให้สอดคล้องกับประกาศของทางราชการ เพื่อให้สามารถควบคุมการระบาดของโรคได้

ทั้งนี้ ตั้งแต่บัดนี้เป็นต้นไป

ประกาศ ณ วันที่ [illegible] พฤษภาคม พ.ศ. ๒๕๖๓

([illegible]สตราจารย์ชาญชัย พานทองวิริยะกุล)
รัก[illegible]การแทนอธิการบดีมหาวิทยาลัยขอนแก่น
