# Supplementary material for: Effects of Bafa Wubu and He-Style Tai Chi exercise training on physical fitness of overweight male university students: A randomized controlled trial
Source: PLoS One. 2024 Jan 19;19(1):e0297117. doi: 10.1371/journal.pone.0297117 (PMC10798526; doi:10.1371/journal.pone.0297117)
Supplement: S2 File — (PDF) [file pone.0297117.s002.pdf]

The Effects of Two Different Types of Tai Chi Exercise Training on Overweight People: A Study on Chinese University Students

Research Protocol

1. Introduction

From 1980 to 2015, the prevalence of overweight in the world from 26.5% to 39.0%; obesity from 7% to 12.5%, and the overweight rate in China from 7.8% to 29.9% (Chooi et al., 2019). Among Chinese college students aged 19-22, the proportion of males who are overweight or obese is 22.7% and that of females is 8.4% (Chen et al., 2020). The problem of overweight or obesity among Chinese college students is still a major problem facing society. Overweight or obesity can affect various functions of the human body, and constitute a major public health threat, and bring a heavy burden to happiness and society (ten Hoor et al., 2017).

In previous studies, a large amount of evidence showed that physical exercise can reduce risk factors related to overweight or obesity and enhance physical fitness (Penedo and Dahn, 2005). Tai Chi is a systematic whole-body movement developed in ancient China (Chengyao et al., 2020). Previous studies have shown that Tai Chi exercise can improve the blood lipids and physical fitness of overweight or obese people. Limitations of previous Tai Chi intervention studies: 1) The research objects are concentrated in adolescents, middle-aged and elderly patients. 2) Simplified Tai Chi or modified Tai Chi is widely used as a means of intervention. 3) Comparative study of Tai Chi and other sports. 4) Intervention research on traditional Tai Chi is rarely involved.

Previous studies have shown the benefits of Tai Chi exercise for obesity and overweight. However, the newly compiled simplified Bafu Wubu Tai Chi and He-style Tai Chi have not yet found research on the effects of obese or overweight people. Tai Chi is an aerobic exercise, but it has never been studied for 30 minutes of continuous exercise to assess heart rate and intensity. Especially the difference in intensity level between Bafu Wubu Tai Chi and He-style Tai Chi. Intervention research on the

influence of traditional and simplified Tai Chi on overweight students is still unclear. Studies comparing the effects of two different styles of Tai Chi intervention have not yet been found.

Based on the above reasons, we plan to use a randomized controlled trial to conduct two different types of Tai Chi training for overweight students. By detecting the changes in blood lipids and physical fitness indicators of overweight practitioners, we will systematically analyze the effect of Tai Chi exercise on overweight students and compare them. The effects of these two types of Tai Chi exercises are expected to obtain objective and scientific evidence, provide a scientific basis for the effect of Tai Chi exercises on overweight student groups. This research on the influence of traditional Tai Chi and simplified Tai Chi on the blood lipids and physical fitness of overweight students will fill the gaps in previous studies.

2. Objectives of the study

Using randomized controlled trials, one is to evaluate the effects of two different Tai Chi exercises on the physical fitness and blood lipids of overweight male college students, and the other is to compare the effects of two different Tai Chi on physical fitness and blood lipids.

3. The research protocol and technical route

3.1 Study design

This study will use a randomized controlled trial (RCT).

3.1.1 Randomization and allocation concealment

The eligible participants will be randomly allocated in a 1:1:1 ratio to either the Bafa Wubu Tai Chi group, tradition He-styles Tai Chi group and control group. The random allocation sequence will be generated using web programming at http://www.randomizer.org by the project manager. The eligible participants will be informed of their allocation result by the project manager via telephone after their baseline information has been assessed.

3.1.2 Blinding

Although it is impossible to blind the participants and exercise coaches due to this

being a non-pharmacological intervention trial. The participants' allocation result will be replaced by using alphabet 'A', 'B' or 'C' in the first blind code and the real meaning of 'A', 'B' or 'C' will be marked in the second blind code. The project manager will declare the real meaning of group 'A', 'B' or 'C' after analysis of all data is completed. (A: The Bafa Wubu Tai Chi group, B: Tradition He-styles Tai Chi group C: control group)

3.2 Participants

Research subjects will be recruited from three universities, Jiaozuo Teachers College, Jiaozuo University and Henan University of Technology, Jiaozuo City, Henan Province, China. These research subjects will be male college students, selected according to China's overweight standard BMI. All participants are interested in Tai Chi activities.

3.2.1 Inclusion criteria

1) Age 18-23 years old

2) Male college students in the first or second year of university

3) Informed consent, voluntary participation

4) $24 \leqslant \text{BMI} < 27.9$ (Chinese overweight standard)

Those who meet the above criteria can be included in the study.

3.2.2 Exclusion criteria

1) Regular long-term Tai Chi athletes

2) Have any sports association members

3) Severe cardiovascular disease or musculoskeletal system disease

4) $\text{BMI}<24$ or $\text{BMI}\geq 28$ (Chinese overweight standard)

Those who meet any of the above criteria will not be included in the study.

3.2.3 Exit criteria

1) Those who can't insist on training;

2) Those who do not practice according to the training requirements;

3) Sudden illness or injury occurred during the test and cannot continue to participate in the training;

4) Those who voluntarily propose to quit the researcher;

5) The control group members practice Tai Chi during the experiment;

6) Those who have adverse events and are not suitable to continue practicing.

3.3 Sample size

The purpose of this study is to evaluate the effect of the newly compiled Bafa Wubu simplification Tai Chi and traditional He-style Tai Chi on the blood lipids and physical fitness of overweight college students. Therefore, using a randomized controlled trial study design (RCT). Since blood lipids are the main outcome variable of the study, however, the previous research literature did not have 3 groups of test subjects similar to this research, similar exercise intensity and similar parameters. Therefore, G Power 3.1.9.4 is used to estimate the sample size. Since the experiment is designed into three groups, select "ANOVA: Fixed effects, omnibus, one-way" in F tests, select "A priori: Compute required sample size" in Analysis, and set the effect size conventions to the maximum value f=0.40, α err prob is set to 0.05, power (1-β err prob) value is set to 0.80, Number of groups is set to 3. The calculation steps are as follows:

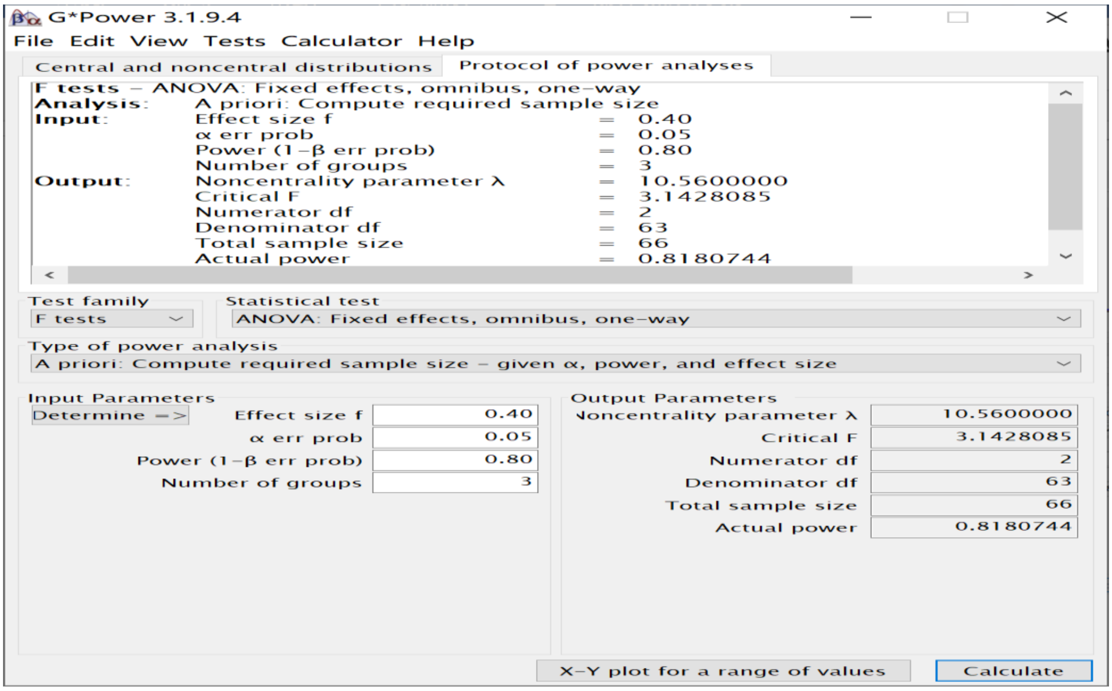

Figure: 4 Sample size calculation.

The total sample size is 66 persons. The sample size is 22 persons per each group.

Drop out = 20% = 4.4 = 5

Therefore, the number of sample size of each group was 27 subjects. The sample size of this study is set to 30 persons per group. The purpose of this setting is to further improve the inspection performance (Power value). Substitute 30 samples per group into the G Power calculation program to derive a higher performance value (Power value). So each group will select 30 sample sizes.

3.4 Intervention method

3.4.1 Bafa Wubu Tai Chi group

The simplified Tai Chi Bafa Wubu Tai Chi (Eight Methods and Five steps) newly compiled by the State Sports General Administration in 2019 is adopted. Instructors who have more than 5 years of teaching experience and have a Tai Chi teaching qualification certificate conduct instructed exercises. Including 16 action content, as shown in the following table:

1) Start up<br>2) Left squeeze; Right squeeze; Left squeeze; Double press<br>3) Right picking potential; Left pressing potential; Left elbow potential; Right holding potential<br>4) Right-handed posture; Left-handed posture; Right squeezed posture; Double press posture<br>5) Left picking position; Right pressing position; Right elbow position; Left holding position<br>6) Progress and control the situation<br>7) Retreat<br>8) Move left and squeeze left; Move left and double press<br>9) Move right and squeeze right; Move right and double press<br>10) Retreat and take the left and right<br>11) Progress controls the situation<br>12) Step right, right elbow, right step, right leaning<br>13) Move left and left elbow position; Move left and hold left position<br>14) Independence<br>15) Cross hands<br>16) Close up

3.4.2 Traditional He-style Tai Chi group

Using the "He-Style Tai Chi Pu" published by the People's Sports Publishing House of China in 2003. Instructors who have more than 5 years of teaching experience and have a Tai Chi teaching qualification certificate conduct instructed exercises. This traditional He-style Tai Chi consists of 72 movements, as shown in the table below.

Start	26) Two steps and three beats	52) Seabed Needle
1) Three pairs of King Kong	27) Qinglong Exploring the Sea	53) Flashback
2) Lazy tie	28) Second kick	54) Like closed like closed
3) Like closed like closed	29) Separate horseshoes and tuck the knees	55) Single whip
4) Single whip	30) Magpies	56) Cloud Hands
5) The leader of the King Kong	31) Harrier stand up	57) High Detective Horse
6) White crane spreads its wings	32) Left and right knee wraps	58) Single swing foot
7) diagonal	33) Cover your hands	59) Slinging finger crotch pounding
8) Pipa momentum	34) Hold your head and push the mountain	60) King Kong
9) Leap and go diagonally	35) Like closed like closed	61) Lazy tie
10) Turn around pipa pose	36) Single whip	62) Right tie seven stars
11) Jump with arms around knees	37) Before and after move	63) Looking back at the painting
12) Fuhu	38) Remainder	64) Huanglong stirring water
13) Finger thumping	39) Mustang split mane	65) Like closed like closed
14) Look at the fist at the bottom of the elbow	40) Jade Girl Shuttle	66) Single whip
15) Fallen Monkey	41) Turn around lazy tie	67) Left tie seven stars
16) White crane spreads its wings	42) Like closed like closed	68) Progressive cross hand
17) diagonal	43) Single whip	69) Regress and Stride Tiger
18) Seabed needle	44) Cloud Hands	70) Double swing feet
19) Flashback	45) Die Cha	71) Move the bow and shoot the tiger
20) Like closed like closed	46) Sweep the legs	72) Three pairs of King Kong
21) Single whip	47) Golden Rooster Independent	Close
22) Cloud Hands	48) Double shaking feet	
23) High Detective Horse	49) Fallen Monkey	
24) Pat your feet left and right	50) White Crane Spreading Wings	
25) Rotate the heel	51) diagonal	

3.4.3 Control group

Because the control group does not do any exercise intervention, during the 12-week experiment, all participants will be asked to keep normal diet and daily routine.

3.4.4 Warm-up and cool-down before and after the intervention

Warming up before intervention can increase the muscle temperature, increase muscle and tendon elasticity, and prevent sports injuries. Cooling exercises after intervention can reduce muscle soreness and quickly restore physical strength. In this study, static stretching will be used for warm-up and cooling content before and after the intervention. The static stretching content comes from the book "Warm-Up and Stretching" written by Ian Jeffreys, MS in 2008. 15 static stretching actions are selected. They are: 1) Rotation of the neck to the right/left. 2) Neck flexion and Neck extension. 3) Stretching the triceps. 4) Stretching the upper back. 5) Stretching the upper back and forearms. 6) Stretching the lower back and sides. 7) Stretching the low back from a seated position. 8) Stretching the hip flexors. 9) Stretching the sides, triceps, and upper back. 10) Stretching the hamstrings. 11) Stretching the hamstrings and lower back. 12) Stretching the hamstrings and hip adductors. 13) Stretching the hamstrings, hip adductors, and lower back. 14) Stretching the hip adductors. 15) Stretching the calves. In the warm-up and cooling process, each stretching action is required to reach the

individual's best state for at least 30 seconds in order to achieve the effect of stretching. Achieve beneficial warm-up before intervention and body recover after the intervention.

3.5 Intervention time

The Bafa Wubu Tai Chi group and the traditional He-style Tai Chi group will receive Tai Chi training 12 weeks, 3 times a week, each course is 60 minutes, including 30 minutes of Tai Chi exercises and 15 minutes of warm-up and cold down. The control group will have 1 health lecture every month, 3 times in total 12 weeks. During the 12-week experiment, all participants will be asked to keep normal diet and daily routine.

3.6 Data collection

Visit 1 (Baseline): Before the test, all participants performed basic personal physical sign evaluation and filled out the personal physical sign evaluation form. The evaluation content included gender, age, body height, body weight, and BMI value.

Visit 2 (Pre-training): All participants will participate in blood lipid assessment (TC, TG, LDL-C, HDL-C), physical fitness assessment (Body mass, BMI, fat percentage, muscle content, flexibility, upper and lower limbs Strength, balance and aerobic endurance).

Visit 3 (Post-training): All participants will be participated in the examination as same as the assessment of visit 2.

3.7 Outcome measurements

3.7.1 Primary outcome measures

1) Body composition

Measures of body composition variables include: body weight, BMI, fat percentage, muscle content.

Measuring instrument: Body composition analyzer (BCA-2Atype). Place of Origin: produced by China Tongfang Health Technology Company (Beijing).

Measuring place: Physical Fitness Testing Center of Tai Chi Sports Center, Jiaozuo City, China. The assessment is conducted by a qualified assessor.

2) **Blood lipid**

Measures of blood lipid variables include: total cholesterol (TC), triglyceride (TG), low-density lipoprotein cholesterol (LDL-C) and high-density lipoprotein

cholesterol (HDL-C).

Measuring instrument: CardioChek PA Blood Analyzer. Place of Origin: USA

Measuring place: The Physical Fitness Testing Center of Jiaozuo People's Hospital, Jiaozuo City, China. Due to the particularity of blood lipid measurement, the measurement and evaluation will be completed by a qualified nurse.

3.7.2 Secondary outcome measures

1) Flexibility

Measurement name: Sit and Reach Test

Measuring instrument: WQ-168 sitting body forward bending tester. Place of Origin: China Beijing Weixin Yiao Technology Development Company.

Measuring place: Physical Fitness Testing Center of Tai Chi Sports Center, Jiaozuo City, China. The assessment is conducted by a qualified assessor.

2) Upper limb strength

Measurement name: Grip strength test

Measuring instrument: Electronic grip strength tester HKD-1881. Place of Origin: Beijing Hongkangda Sports Technology Company produces.

Measuring place: Physical Fitness Testing Center of Tai Chi Sports Center, Jiaozuo City, China. The assessment is conducted by a qualified assessor.

3) Lower limb strength

Measuring instrument: Wall squat test (Vaegter et al., 2019).

The wall squat test is a simple test of lower-body muscle strength and endurance. The test requires participants to sit while leaning back against the wall. Need flat non-slip floor, smooth wall, and stopwatch.

Measuring place: Physical Fitness Testing Center of Tai Chi Sports Center, Jiaozuo City, China. The assessment is conducted by a qualified assessor.

4) Balance

Static balance measuring: The Balance Error Scoring System (BESS). A previous research report pointed out that the use of BESS to test static balance has high reliability between measurers (within-class correlation coefficient = .78 to .96), and fairness of validity is good (r = .42 to .79) (Riemann et al., 1999), so this method

is used for static balance assessment.

Dynamic balance measuring: The Star Excursion Balance Test (SEBT) (Gribble and Hertel, 2003). Previous research reports pointed out that the use of SEBT to test adult dynamic balance has high reliability between measurers (within-class correlation coefficient = .78 to .96) (Hertel et al., 2000), so this method is used for dynamic balance assessment.

Measuring place: Physical Fitness Testing Center of Tai Chi Sports Center, Jiaozuo City, China. The assessment is conducted by a qualified assessor.

5) **Aerobic endurance**

Aerobic endurance Measuring: The six-minute walk test (6MWT) (Eden et al., 2018)

Measurement place: Standard 800-meter track of Tai Chi Sports Center in Jiaozuo City, China. The assessment is carried out by qualified assessors.

3.7.3 Safety indicators

The side effects and adverse events that occurred during the study were recorded, and the reasons were analyzed. The incidence of adverse events in each group was used as the evaluation index. The calculation formula of the incidence of adverse events is as follows:

The incidence rate of adverse events % = (number of adverse events/total number of cases in this group) ×100%

3.8 Data management and analysis

3.8.1 Data management

The research report form is filled in by the research object and the result evaluation personnel. The research report form consists of an activity log sheet, a blood lipid measurement sheet, a body composition measurement sheet, a physical fitness measurement sheet, and an adverse event record sheet. After the completed research report sheet is reviewed by the project manager, the data entry clerk is organized to enter the data and Unified management of the original table.

3.8.2 Data analysis

Data will be collected by means of Excel 2019, statistical analysis will be carried

out using the IBM SPSS version 26.0 (IBM Corporation, NY, USA). Data will be present as mean ± standard deviation (SD), and 95% confidence interval (CI) adopted. A normality study will be conducted and further descriptive statistics will be carried out. The Shapiro-Wilk test will be used to test whether the data conform to a normal distribution. If the parameters are normally distribution, the one-way ANOVA test will be used to compare the three groups, the independent t-test will be used for two groups comparison, and the Paired t-test will be used to compare the results before and after training within the group. If the parameter is Non-normally distribution, the Kruskal Wallis test will be used to compare the three groups, the Mann-Whitney u test will be used for two groups comparison, and the Wilcoxon rank test will be used to compare the results before and after training within the group. The level of statistical significance will be set at $p<0.05$.

3.9 Quality control

1) Quality control of the informed consent of volunteers: Before signing the informed consent form, extensive education and communication should be carried out by the volunteer participants, so that the volunteers fully understand the content of the informed consent form.

2) Volunteer recruitment quality monitoring: strictly screen, all volunteers according to the inclusion and exclusion criteria.

3) Quality monitoring of random control and blind method: The project manager randomly grouped qualified subjects according to the number of the screener according to the random grouping sequence, properly kept the blind bottom, and unbind after the data analysis is over.

4) Quality control of the intervention process: The project manager supervises the coaching and training process of the coach, and the coach should supervise and guide the training process of the volunteers of the intervention group.

5) Quality monitoring of statistical analysis: The measurement data must be clearly filled in the research report form. Those whose results are zero and those that cannot be detected in the test should be indicated by corresponding symbols and cannot be vacated in order to distinguish them from missing values. If there is a missing value, look up

the original data, and fill it in for missing; if there is no record in the original data, notify the subject to recheck immediately; if the missing value affects the resulting judgment, the case is eliminated.

6) Data management quality control: The list of documents that the project manager must keep is checked one by one. If there are any missing items, the relevant personnel shall be required to fill in them.

Technical route

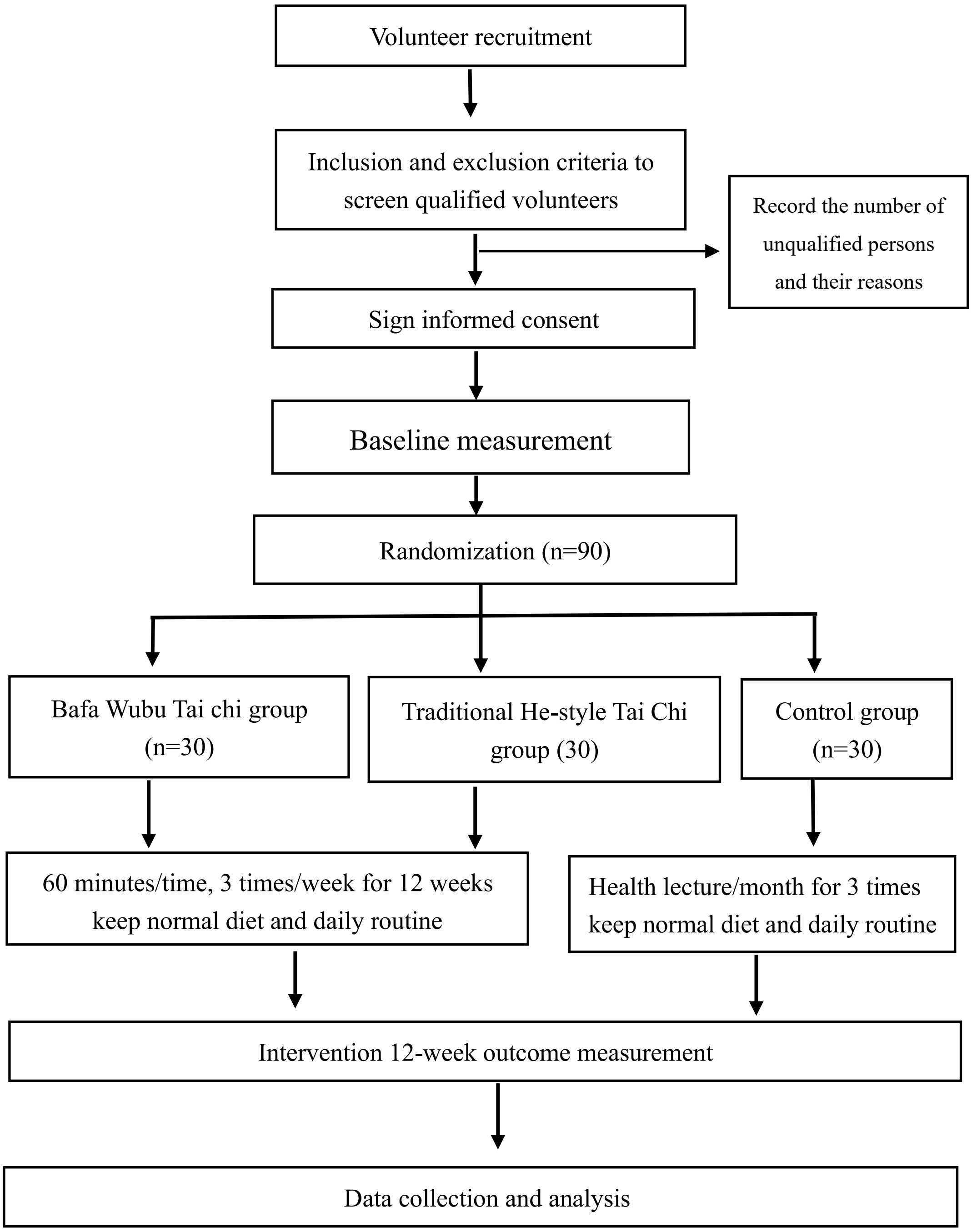

Reference

Chen, Y., Liu, X., Yan, N., Jia, W., Fan, Y., Yan, H., Ma, L., & Ma, L. (2020). Higher academic stress was associated with increased risk of overweight and obesity among college students in China. *International Journal of Environmental Research and Public Health*, *17*(15), 1–12.

Chengyao, G., Xiang, G., Xie, L., Liu, Z., Zhang, X., Wu, Q., Li, S., & Wu, Y. (2020). Effects of Tai Chi training on the physical and mental health status in patients with chronic obstructive pulmonary disease: A systematic review and meta-analysis. *Journal of Thoracic Disease*, *12*(3), 504–521.

Chooi, Y. C., Ding, C., & Magkos, F. (2019). The epidemiology of obesity. *Metabolism: Clinical and Experimental*, *92*(2019), 6–10.

Eden, M. M., Tompkins, J., & Verheijde, J. L. (2018). Reliability and a correlational analysis of the 6MWT, ten-meter walk test, thirty second sit to stand, and the linear analog scale of function in patients with head and neck cancer. *Physiotherapy Theory and Practice*, *34*(3), 202–211.

Gribble, P. A., & Hertel, J. (2003). Considerations for normalizing measures of the Star Excursion Balance Test. *Measurement in Physical Education and Exercise Science*, *7*(2), 89–100.

Hertel, J., Miller, S. J., & Denegar, C. R. (2000). Intratester and intertester reliability during the star excursion balance tests. *Journal of Sport Rehabilitation, 9*(2), 104–116.

Penedo, F. J., & Dahn, J. R. (2005). Exercise and well-being: A review of mental and physical health benefits associated with physical activity. *Current Opinion in Psychiatry, 18*(2), 189–193.

Riemann, B. L., Guskiewicz, K. M., & Shields, E. W. (1999). Relationship between clinical and forceplate measures of postural stability. *Journal of Sport Rehabilitation, 8*(2), 71–82.

ten Hoor, G. A., Kok, G., Peters, G. J. Y., Frissen, T., Schols, A. M. W. J., & Plasqui, G. (2017). The Psychological Effects of Strength Exercises in People who are Overweight or Obese: A Systematic Review. In *Sports Medicine* (Vol. 47, Issue

10, pp. 2069–2081). Springer International Publishing.

Vaegter, H. B., Lyng, K. D., Yttereng, F. W., Christensen, M. H., Sørensen, M. B., & Graven-Nielsen, T. (2019). Exercise-Induced Hypoalgesia After Isometric Wall Squat Exercise: A Test-Retest Reliabilty Study. *Pain Medicine*, *20*(1), 129–137.
