# Supplementary material for: Effects of Bafa Wubu and He-Style Tai Chi exercise training on physical fitness of overweight male university students: A randomized controlled trial
Source: PLoS One. 2024 Jan 19;19(1):e0297117. doi: 10.1371/journal.pone.0297117 (PMC10798526; doi:10.1371/journal.pone.0297117)
Supplement: S5 File — (PDF) [file pone.0297117.s005.pdf]

# Henan Provincial Department of Education

# 河　南　省　教　育　厅

**Key scientific research project plan of colleges and universities in Henan Province**
**Project notification**

## 河南省高等学校重点科研项目计划

## 立 项 通 知

焦作师范高等专科学校:

你单位申报的下列研究课题，经专家评审、省教育厅审核，已列为河南省高等学校重点科研项目计划，并以 教科技〔2021〕383号 文件批准下达。现通知如下:

项目编号：22B890001

项目名称：两种不同类型太极拳训练对超重或肥胖人群的影响：一项对大学生的研究

项目负责人：牛炎涛 **Niu Yantao**

项目研究期限：2022年01月01日--2023年12月31日

项目组成员：

| 排序 | 姓名 | 性别 | 单位 |
|---|---|---|---|
| 2 | 刘巧芳 | 女 | 焦作师范高等专科学校 |
| 3 | 杨惠晓 | 女 | 焦作师范高等专科学校 |
| 4 | 孙红元 | 男 | 焦作师范高等专科学校 |
| 5 | 胡精超 | 男 | 河南理工大学 |
| 6 | 靳贤圣 | 男 | 焦作师范高等专科学校 |
| 7 | 程峰 | 男 | 焦作师范高等专科学校 |
| 8 | 蔺丽萍 | 女 | 焦作师范高等专科学校 |
| 9 | 孟祥港 | 男 | 焦作师范高等专科学校 |
| - | - | - | - |
| - | - | - | - |

（项目组共 9 人）

河南省教育厅 科研管理专用章

2021年10月22日
