# Supplementary material for: Effects of Bafa Wubu and He-Style Tai Chi exercise training on physical fitness of overweight male university students: A randomized controlled trial
Source: PLoS One. 2024 Jan 19;19(1):e0297117. doi: 10.1371/journal.pone.0297117 (PMC10798526; doi:10.1371/journal.pone.0297117)
Supplement: Attachment — Submitted filename: 20230907_review_comments.docx [file pone.0297117.s006.docx]

Thanks for inviting me to review this wonderful work conducted by Rajapon and the rest of the team. This RCT examined the effects of two types of tai chi exercise on improving different physical fitness parameters in overweight university male students in mainland China during the COVID-19 pandemic. The authors identified a very interesting research gap in the current literature that there is a lack of head-to-head comparison between different types of tai chi on improving physical health. This trial was conducted of standard, and the conclusion was well supported by the results.

I have a few minor suggestions for the authors, listed as following:

1. Since the study participants are all male, I suggest the authors to indicate this in the study title.
2. In the abstract line 41 and 42, the improvements in sitting and reaching, wall squat, and 6-minute walk should be positive numbers? The minus symbol could be a mistake? Please check.
3. The study did not indicate which outcome is the primary outcome of the study, which is one of my major concerns, which is related to my next concern – sample size calculation.
4. In line 95 to 98, the authors briefly mentioned the method of sample size calculation, but the information about the effect size of the intervention(s) on the primary outcome, α, and power were missing. Were drop-out rates considered during the sample size calculation? Since this RCT was completed anyway, I suggest the authors to perform a power analysis to retrospectively investigate the power under the current sample size.
5. In the results section, were there any improvements in body weight and BMI? As the study participants were all overweight, this will be of the readers' interest to know.

Despite my concerns regarding the sample size calculation and primary outcome identification, I believe that this study offers valuable insights and innovative ideas to the field. I would suggest making minor revisions before publishing this work.
