# Supplementary material for: Effects of Bafa Wubu and He-Style Tai Chi exercise training on physical fitness of overweight male university students: A randomized controlled trial
Source: PLoS One. 2024 Jan 19;19(1):e0297117. doi: 10.1371/journal.pone.0297117 (PMC10798526; doi:10.1371/journal.pone.0297117)
Supplement: Attachment — Submitted filename: Response to Reviewers.docx [file pone.0297117.s007.docx]

**Response to Reviewers**

Thank you to the reviewers and editor for the really helpful and constructive comments and providing us with the opportunity to make revisions to the manuscript to be considered for publication. We hope that by addressing the significant amount of feedback and comments from the reviewers, that you feel we have strengthened the manuscript.

On the revised manuscript, Editor’s revisions are highlighted in red, Reviewer #1’s is highlighted in yellow, Reviewer 2’s is highlighted in blue. Responses to all the comments are in bold underneath each comment below.

**Editor:** General comments

1. Please ensure that your manuscript meets PLOS ONE's style requirements, including those for file naming.

**Thank you for this comment. We have now checked according to the style requirements of PLOS ONE, including the file naming requirements, to ensure compliance with the journal's requirements.**

2. Thank you for stating the following in the Acknowledgments Section of your manuscript:

"We wish to acknowledge all participants and staff who participated in this research activity and the support from Jiaozuo Normal University. We also wish to acknowledge the technical experts from the national physical health testing centre of Jiaozuo Tai Chi Sports Center for providing testing equipment services. This work was supported by the 2022 Key University Research Project of the Department of Education of Henan Province, China grant number 22B890001."

We note that you have provided funding information that is not currently declared in your Funding Statement. However, funding information should not appear in the Acknowledgments section or other areas of your manuscript. We will only publish funding information present in the Funding Statement section of the online submission form.

Please remove any funding-related text from the manuscript and let us know how you would like to update your Funding Statement. Currently, your Funding Statement reads as follows:

"The author(s) received no specific funding for this work."

Please include your amended statements within your cover letter; we will change the online submission form on your behalf.

**We greatly appreciate the details of the reviewers for the constructive feedback. We have now removed the funding information from the acknowledgments section (p.1, line 27, p.21, line 356), and added the funding information to the cover letter.**

3. We note that you have stated that you will provide repository information for your data at acceptance. Should your manuscript be accepted for publication, we will hold it until you provide the relevant accession numbers or DOIs necessary to access your data. If you wish to make changes to your Data Availability statement, please describe these changes in your cover letter and we will update your Data Availability statement to reflect the information you provide.

**Thank you for this comment. Yes, we have stated that we will provide repository information for the data upon acceptance.**

4. Please include captions for your Supporting Information files at the end of your manuscript, and update any in-text citations to match accordingly. Please see our Supporting Information guidelines for more information: http://journals.plos.org/plosone/s/supporting-information.

**We greatly appreciate the details of the reviewers for the constructive feedback. We have now added titles to supporting information files at the end of the manuscript (p.26, line 510-515),** **and update all in-text citations accordingly (p.4, line 91, p.6, line 119-120, p.7, line 141, p.11, line 216).**

**Reviewer #1:** Comments

Reviewer #1: A three-arm randomized controlled trial was conducted which aimed to compare the effects of 12 weeks of Bafa Wubu Tai Chi (BW-TC) to traditional He-style Tah Chi (TH-TC) to control on physical fitness. The conclusions are unclear.

**Thank you to the reviewer for the really helpful and constructive comments and providing us with the opportunity to make revisions to the manuscript to be considered for publication. We hope that by addressing the significant amount of feedback and comments from the reviewers, that you feel we have strengthened the manuscript.**

**Major revisions:**

1- Line 193-198: The ANOVA should be used to test the interaction effect of group by time. If the interaction effect is significant, provide an interpretation of the results, but do not test main effects because the tests for main effects are uninteresting in light of significant interactions. If interaction effects are non-significant, drop the interaction effects from the model and test the main effects. Determining which results to present when testing interactions is often a multi-step process. Specify the statistical method used for step-down testing when a significant interaction effect was observed.

**We greatly appreciate the details of the reviewer for the constructive feedback. We have now corrected and stated the statistical analysis methods** (**p.10, line 203-211**).

2- Line 197-198: If the interaction test is conducted, the dependent t-tests are not necessary.

**We greatly appreciate the details of the reviewers for the constructive feedback. We have now removed the statements related to the t-test (p.10, line 210-211).**

3- More clearly interpret the results associated with significant interaction effects.

**We greatly appreciate the details of the reviewer for the constructive feedback. We now have a clear explanation of the statistical analysis method and significant interactions (p.10, line 203-211).**

**Minor revisions:**

1- Abstract: Specify the type of statistical summary values represented by -3.11/-4.52 cm, -27.56/-36.85 s, and -22.93/-63.22 m.

**We greatly appreciate the details of the reviewers for the constructive feedback. We now have a revised type of statistical summary values represented in summaries (p.2, line 40-42).**

2- The statistical term for average is mean.

**We greatly appreciate the details of the reviewers for the constructive feedback. We have now replaced average with mean (p.2, line 41,43).**

3- Lines 95-98: Provide complete details for estimating the sample size. The power calculation should include: (1) the estimated outcomes in each group; (2) the α (type I) error level; (3) the statistical power (or the β (type II) error level); (4) the target sample size and (5) for continuous outcomes, the standard deviation of the measurements. (6) The statistical testing method used.

**We greatly appreciate the details of the reviewer for the constructive feedback. We have now provided full details of the sample size calculation as suggested by the reviewers (p.5, line 98-104).**

4- Line 115: If block randomization was used, indicate the block size.

**We greatly appreciate the details of the constructive feedback provided to us by the reviewers.**

**We are not using block randomization.**

5- Line 190: Typographical error: presented.

**We greatly appreciate the details of the reviewers for the constructive feedback. Now we have replaced present with presented (p.10, line 199).**

6- Line 191: Specify the descriptive statistics. Indicate if the variables were checked for a normal distribution. Normally distributed variables are typically summarized using means and standard deviations while non-normally distributed variables are summarized with medians, first, and third quartiles.

**We greatly appreciate the details of the reviewer for the constructive feedback. We have now added a statement in the Statistical Analysis Methods section about the test method for variables that are normally distributed (p.10, line 200-201).**

7- Line 192: Replace “portray” with “summarize.”

**We greatly appreciate the details of the reviewers for the constructive feedback. Now we have replaced portray with summarize (p.10, line 202).**

8- Lines 207-210: To improve clarity, rearrange the presentation of results for time-by-group interaction effects. State those that were significant first.

**We greatly appreciate the details of the reviewer for the constructive feedback. We have now rearranged the results for the time-by-group interaction effect and stated the important ones first, as suggested by the reviewers (p.11-12, line 223-228).**

9- P-values never equal zero; express small p-values as < 0.001.

**We greatly appreciate the details of the reviewers for the constructive feedback we provided.** **We have now revised the *p*-value to express the smaller *p*-value as < 0.001** **(p.15, line 247, 248, 253, 256).**

10- Insert the tables where they should appear within the text.

**We greatly appreciate the details of the reviewer for the constructive feedback. Now we have inserted the table into the text where it should appear (p.11, Table 1; p.13, Table 2; p.14, Table 3; p.16, Table 4.)**

**Reviewer #2:** Comments

Reviewer #2: Thanks for inviting me to review this wonderful work conducted by Rajapon and the rest of the team. This RCT examined the effects of two types of tai chi exercise on improving different physical fitness parameters in overweight university male students in mainland China during the COVID-19 pandemic. The authors identified a very interesting research gap in the current literature that there is a lack of head-to-head comparison between different types of tai chi on improving physical health. This trial was conducted of standard, and the conclusion was well supported by the results.

**Thank you to the reviewer for the really helpful and constructive comments and providing us with the opportunity to make revisions to the manuscript to be considered for publication. We hope that by addressing the significant amount of feedback and comments from the reviewers, that you feel we have strengthened the manuscript.**

I have a few minor suggestions for the authors, listed as following:

1. Since the study participants are all male, I suggest the authors to indicate this in the study title.

**We greatly appreciate the details of the reviewer for the constructive feedback. Now we have indicated this in the study title (p.1, line 1).**

2. In the abstract line 41 and 42, the improvements in sitting and reaching, wall squat, and 6-minute walk should be positive numbers? The minus symbol could be a mistake? Please check.

**We greatly appreciate the details of the reviewer for the constructive feedback.** **Now we have checked and corrected the evaluation metric improvement effect to be a positive number** **(p.2, line 41-42).**

3. The study did not indicate which outcome is the primary outcome of the study, which is one of my major concerns, which is related to my next concern – sample size calculation.

**We greatly appreciate the details of the reviewer for the constructive feedback. The reason why the main results of this study were not explicitly specified is that these results are part of our earlier research. The physical fitness is our primary outcome. Our sample size calculation was conducted using the G*Power software.**

4. In line 95 to 98, the authors briefly mentioned the method of sample size calculation, but the information about the effect size of the intervention(s) on the primary outcome, α, and power were missing. Were drop-out rates considered during the sample size calculation? Since this RCT was completed anyway, I suggest the authors to perform a power analysis to retrospectively investigate the power under the current sample size.

**We greatly appreciate the details of the reviewer for the constructive feedback. We have now retrospectively examined the statistical power under the current sample size, considering effect size, α, statistical power, and dropout rates. Additionally, we have provided further clarification regarding the rationale behind our sample size calculation (p.5, line 96-104).**

5. In the results section, were there any improvements in body weight and BMI? As the study participants were all overweight, this will be of the readers' interest to know.

**We greatly appreciate the details of the reviewer for the constructive feedback. We have now added statements about improvements in weight and BMI in the results section (p.15, line 260-266),** **and added a citation of our previous study results and presented it in the References section (p.24, line 452-455).**
