# Supplementary material for: Effects of Bafa Wubu and He-Style Tai Chi exercise training on physical fitness of overweight male university students: A randomized controlled trial
Source: PLoS One. 2024 Jan 19;19(1):e0297117. doi: 10.1371/journal.pone.0297117 (PMC10798526; doi:10.1371/journal.pone.0297117)
Supplement: Attachment — Submitted filename: Response to Reviewers.docx [file pone.0297117.s008.docx]

**Response to Reviewers**

Thank you to the reviewers and editor for the really helpful and constructive comments and providing us with the opportunity to make revisions to the manuscript to be considered for publication. We hope that by addressing the significant amount of feedback and comments from the reviewers, that you feel we have strengthened the manuscript.

On the revised manuscript, Reviewer #1’s is highlighted in yellow. Responses to the comments are in bold underneath each comment below.

**Additional Editor Comments:**

Please address the data analysis concerns as indicated by Reviewer#1 and modify your results and discussion accordingly.

**Thank you for this comment. We have now edited the data analysis presentation again based on the reviewers' comments.**

**Reviewer #1:** Comments

**Major revisions:**

The authors failed to make the distinction between interaction effects and main effects. Interaction effects include more than one variable. For instance time-by-group is an interaction effect. But the time effect alone is not an interaction effect; it is a main effect. I recommend that a qualified statistician be consulted to conduct these analyses and appropriately distinguish been interaction and main effects. Revise the wording in lines 203-211 (tracked changes version) to indicate that the standard practice for testing interactions has been conducted.

Standard Practice for testing interaction effects: If the interaction effect is significant, provide an interpretation of the results, but do not test main effects because the tests for main effects are uninteresting in light of significant interactions. If interaction effects are non-significant, drop the interaction effects from the model and test the main effects. Determining which results to present when testing interactions is often a multi-step process.

**Thank you to the reviewer for the really helpful and constructive comments and providing us with the opportunity to make revisions to the manuscript to be considered for publication. We hope that by addressing the significant amount of feedback and comments from the reviewers, that you feel we have strengthened the manuscript. We have now consulted with qualified statistics teachers to guide us in editing the statistical analysis methods again** (**p.10, line 203-210**).
